# Preference For Cervical Cancer Education: A Multisite Cross-Sectional Survey of Female Senior High School Students in Ghana

**DOI:** 10.1007/s13187-023-02325-5

**Published:** 2023-06-16

**Authors:** Ama Gyamfua Ampofo, Allison W. Boyes, Lisa J. Mackenzie

**Affiliations:** 1 Health Behaviour Research Collaborative, School of Medicine and Public Health, College of Health, Medicine and Wellbeing, The University of Newcastle, Callaghan, NSW 2308 Australia; 2 Equity in Health and Wellbeing Research Program, Hunter Medical Research Institute, New Lambton Heights, NSW Australia

**Keywords:** Uterine Cervical Neoplasms, Students, Education, Preferences, Adolescents, Ghana

## Abstract

Cervical cancer incidence continues to rise in Ghana. To enhance knowledge and prevention of cervical cancer among young people in Ghana, there is a need to better understand their education preferences. The study aimed to describe female senior school students’ preferences for receiving cervical cancer education. A cross-sectional survey of students from 17 schools in the Ashanti Region of Ghana assessed the strength of preference for receiving cervical cancer education from a range of sources, settings and delivery mediums. Of the 2400 participants (aged 16–24 years), the majority endorsed doctors (87%, 95%CI: 85–88%), nurses (80%, 95%CI: 78–82%) and credible health organisations (78%, 95%CI%: 76–79%) as their preferred source of education, and hospitals 83% (95%CI: 81–84%) as the preferred setting. Nearly all students (92%) endorsed at least three cervical cancer education delivery mediums, with at least three quarters endorsing television (78%, 95%CI: 77–80%), one-on-one health consultation in-person or online (77%, 95%CI: 75–79%; 75%, 95%CI: 73–77%), and health information websites (75%, 95%CI: 73–77%). Findings suggest that cervical cancer education efforts among female senior school students in Ghana should consider the use of a range of more resource-intensive individualised approaches to low-cost anonymous, generic approaches from credible sources and institutions.

**Supplementary Information:**

The online version contains supplementary material available at 10.1007/s13187-023-02325-5.

## Introduction

Cervical cancer is increasing in Low-Middle Income Countries (LMICs). Of the 604,127 new cases of cervical cancer and 342,831 deaths linked to cervical cancer [1], about 84% occurred in LMICs including Ghana [2]. It is projected that cervical cancer incidence will increase by 25% over the next 10 years in LMICs. In Ghana, cervical cancer cases will rise from 2797 in 2020 [1] to 5940 by 2025 [3]. However, the disease is highly preventable. Primary prevention strategies include HPV vaccination and modifiable behaviours such as safe sexual practices and not smoking [1]. Secondary prevention involves cervical cancer screening. These strategies implemented throughout adolescence and early adulthood are effective in reducing cervical cancer morbidity and mortality [1]. Enhanced primary and secondary prevention programs have reduced cervical cancer incidence in developed countries such as Australia, where age-standardised incidence dropped from 14 per 100,000 women in 1982 to 6.9 per 100,000 women in 2011, and is projected to drop to fewer than four new cases per 100,000 women by 2028 [4].

Adolescence and young adulthood are critical periods for providing health education about cervical cancer including risk reduction, HPV vaccination and cervical cancer screening. Targeting cervical cancer education to adolescents and young adults is important because they; a) are in the active learning stage [5] b) are underinformed about cervical cancer [6] c) are likely to engage in sexual behaviours that may increase risk of exposure to HPV infection and HPV-related cervical cancer including smoking, early sexual debut and unprotected sex [7] and d) fall within the target age bracket (9–26 years) for HPV vaccination and cervical cancer screening (21 – 65 years) [8].

According to previous studies, young people in Low-Middle-Income Countries (including Ghana and Nigeria) have lower vaccination (< 10%) [9] and screening uptake (< 20%) [10] than those in High Income Countries [11]. Misinformation and lack of information are major challenges to uptake of HPV vaccination [11] and cervical cancer screening services [10]. For example, in Kenya, misinformation about the side effects and efficacy of the HPV vaccines contributed to the low uptake (33% of adolescents received first dose) of the recently initiated vaccination programme [12]. In Sub-Saharan Africa, lack of awareness about susceptibility to cervical cancer and screening locations, and misinformation about the effectiveness of screening for detecting cervical cancer led to poor uptake of screening among age-eligible adolescents and young women [10]. These findings clearly highlight that considerable improvement in health education for cervical cancer is needed.

According to the World Health Organisation, health education is any combination of learning experiences designed to improve health by increasing knowledge, influencing attitudes and motivation, and developing life skills and confidence [13]. Health education strategies that have been trialled for promoting cancer prevention among young people include peer-led, school-based education and community-/family-based [14]. A recent systematic review found that school-based education did not improve attitudes toward HPV vaccination and vaccine uptake among students [15]. However, while a multicomponent approach (including a combination of school-based, community and parental involvement) has been recommended for promoting health in young people [16], a tailored approach may enhance their impact in various settings and health conditions [14]. Understanding adolescents’ preferences for education is important for tailoring appropriate interventions.

Of the few studies exploring adolescents preferences for sexual and reproductive health education, it has been found that some young people prefer personal sources (i.e., family, friends) and health care professionals for sexual and reproductive health information [17], whilst mass media (i.e., television, radio and the internet) and schools have been reported as the most preferred medium [18] and settings for sexual and reproductive health (SRH) information [18], respectively. These studies provide some information about young peoples’ education preference in a related health condition. However, majority of these studies were conducted in high income countries including Europe and America, and explored a limited range of preferences for settings and mediums of education. Sociocultural considerations were also not included. There is the need to increase research about education preferences in young people from low-to-middle income settings.

Two previous studies have reported preferences for cancer-specific health education among adolescents in America. A qualitative study involving twenty five focus groups of high school students, found that the most preferred sources, settings and medium of cancer education were health experts and cancer survivors, schools and online videos, and face-to-face presentations respectively [19]. A quantitative study of 233 high school students reported that over 85% preferred educational games for learning about cancer [20]. Surprisingly, while online resources were highly endorsed in these studies, students identified it as a potential source of misinformation [19]. However, internet accessibility and cost may affect the feasibility of online education in low-income settings and warrants further research into practical and setting-specific approaches.

Empowering young people about cancer prevention requires investigating specific preference needs and settings [14]. While there is some evidence to guide the development of cancer education among young people [14, 19, 20], culturally tailored interventions are limited, and this hinders generalisability and translation of findings [14]. Of the few studies about cancer education preferences, considerable gaps were identified including lack of education preference needs on the different cancer types, limited studies on low-income settings, less rigorous convenience sampling technique, and small sizes [19, 20].

There is also a need to assess preferences for other culturally relevant education sources (i.e., religious and traditional), settings (i.e., home and community) and mediums (i.e., mass media, telephone, different online formats etc.) of education. To the author’s knowledge, no study has comprehensively assessed various cervical cancer education preferences among young people. The study aimed to describe preferences for delivery sources, settings and mediums of cervical cancer education among female senior students in Ghana.

## Methods

### Study Design, Setting and Period

This study is part of a larger cross-sectional study which assessed knowledge and attitudes about cervical cancer and HPV prevention and the prevalence of cervical cancer and HPV risk factors in the Ashanti region of Ghana. The study took place in seventeen accredited public senior high schools between February and July 2021. The study protocol and aspects of the study findings have been reported in detail elsewhere [21].

### Participants

Two thousand four hundred female students were selected from eligible mixed and single-sex (all girls) public senior high schools (SHS). Students who were 16 years and above, and able to obtain parent/guardian consent were eligible to participate [21].

### Procedure

Schools were randomly selected by an independent statistician and eligible students were recruited by research assistant [21]. Students completed questions about sources, settings and mediums of cervical cancer education delivery preference, which was part of larger anonymous pen-and-paper survey assessing demographic characteristics. Students were informed that they could leave the survey items blank. Neither heads of schools nor teachers knew which students completed the surveys. For students under 18 years, written parental/guardian consent was obtained before surveys were conducted.

### Measures

Participants were asked to indicate the extent (on a 4-point Likert scale; strongly disagree, disagree, agree and strongly agree) to which they preferred to receive cervical cancer education from the following:

1. *Sources (10 items):* nurses, doctors, pharmacists, friends, classmates, teachers, family members (e.g. father, mother, sisters, brothers, etc.), credible health organisations (e.g. World Health Organisation), traditional leaders (e.g. queen mothers, chiefs) and religious leaders (e.g. pastor, Imam).
2. *Settings (6 items):* school (during and after class), hospital, pharmacy, place of worship, marketplace and home.
3. *Mediums (12 items)*: face-face group presentation, one-on-one health talk, booklet, live interactive online presentation, recorded online presentation, social media, health information website, online consultation with a health professional, telephone calls, text message, radio, television.
4. *Free-text comments:* For any of the preference domains, participants had the option to provide additional information.

#### Demographic Characteristics

Measures included age, grade (SHS1/SHS2/SHS3), ethnicity (Akan/Northerners/Ewe/Guans, Ga Adangbe/other), residential location (City/Town/Village), Enrolment status (Boarders/ Non boarders), Program of study (Science/General Arts/Business/Visual Arts/ Home economics/Agricultural science), religion (Christianity/Islam/other) and relationship status (Single/dating/other).

### Data Analysis

Data were analysed using Stata statistical software version 16.1 (StataCorp, College Station, Tx). Descriptive analysis was presented as means, standard deviations, median (25^th^, 75^th^ percentile) and percentages. Due to small cell count, the following categorical variables were recoded: Relationship (Single/ Dating or other) and Religion (Christianity/Islam or other). The response options (strongly disagree, disagree, agree and strongly agree) for the outcomes (i.e., sources, settings and mediums of information) were also combined as “most preferred” (Strongly agree and Agree) and “least preferred” (Strongly disagree and Disagree). The proportion of participants who endorsed “Strongly agreed and Agreed” to all outcomes were estimated. Confidence interval and significant P-value were set at 95% and less than 0.05, respectively.

#### Sample Size Estimation

As part of the larger study [21], a sample size of 2400 was estimated a-priori for a cluster randomised design. This sample size was used in the current study.

## Results

Data were collected from at least 140 students per school from 17 schools, resulting in a total of 2400 participants. The mean age of participants was 17.32 years (SD = 1.22; range 16–24), more than 70% of students belonged to the Akan tribe and 46% were in SHS level one. The majority (70%) of students were boarders, and 55% were enrolled in General/Visual Arts. More than 90% were Christians and 71% were single. The sociodemographic characteristics of participants are presented in Table 1.

**Table 1:** Participant characteristics, *N* = 2,400

Variables	*N*^a^ (%)
*Age, Mean (SD)* = 17.32 (1.22); *Median* = *17;* *25*^*th*^* percentile* = *16; 75*^*th*^* percentile* = *18*	
16 years	710 (30%)
17 years	771(32%)
18–24 years	990 (38%)
*Grade*	
SHS 1	1,107 (46%)
SHS 2	573 (24%)
SHS 3	720 (30%)
*Ethnicity*	
Akan	1,860 (77%)
Northerners	300 (13%)
Ewe/Guans	121 (5%)
Ga-Adangbe/other	115 (5%)
*Residential location*	
City	1,089 (46%)
Town	1,108 (47%)
Village	156 (7%)
*Enrolment status*	
Boarders	1,821(76%)
Non-boarders	560 (24%)
*Program of study*	
Science	287 (12%)
General /Visual arts	1,320 (55%)
Business	138 (6%)
Home economics	584 (24%)
Agricultural science	59 (2%)
*Religion*	
Christianity	2,230 (93%)
Islam	153 (6%)
Other	13 (1%)
*Relationship status*	
Single	1,689 (71%)
Dating	648 (27%)
Other	56 (2%)

^a^May not add up to 2,400 due to missing data

### Preferences for Sources of Cervical Cancer Education

**Fig. 1:**
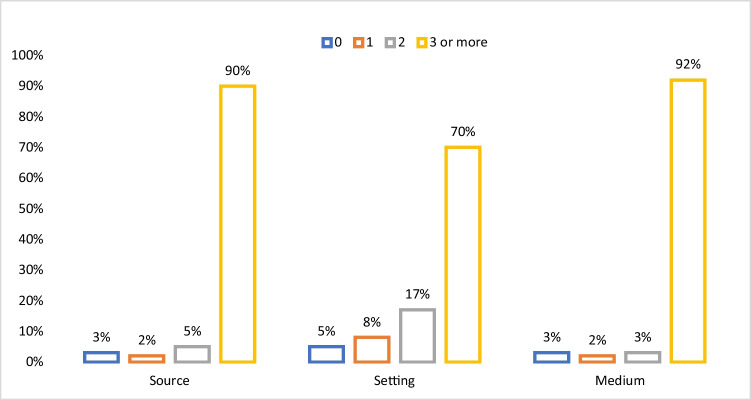
Percentage of students endorsing 0, 1, 2 and 3 + preferred cervical cancer education sources, settings and mediums

Students most preferred sources of cervical cancer education were doctors (87%), nurses (80%), and credible health organisations (78%). The least preferred sources of cervical cancer education were classmates (25%), friends (26%) and traditional leaders (36%) (see Table 2). Almost all students endorsed at least three sources of education items (see Fig. 1).

**Table 2:** Students’ cervical cancer education preferences

Sources				
Rank	Items	*N* ^*a*^ (%)	Denominator	95%CI
1	Doctor	2080 (87%)	2394	(85–88%)
2	Nurse	1912 (80%)	2391	(78–82%)
3	Credible health organization	1867 (78%)	2399	(76–79%)
4	Family members	1506 (63%)	2395	(61–65%)
5	Pharmacist	1483 (62%)	2385	(60–64%)
6	Teachers	1330 (56%)	2394	(54–58%)
7	Religious leaders	960 (40%)	2386	(38–42%)
8	Traditional leaders	857 (36%)	2394	(34–38%)
9	Friends	624 (26%)	2386	(24–28%)
10	Classmates	599 (25%)	2388	(23–27%)
Settings				
Rank	Items	*N* ^*a*^ (%)	Denominator	95%CI
1	Hospital	1977 (83%)	2393	(81–84%)
2	School	1600 (67%)	2400	(65–69%)
3	Home	1440 (60%)	2394	(58–62%)
4	Pharmacy	1359 (57%)	2389	(55–59%)
5	Place of worship	878 (37%)	2394	(35–39%)
6	Market place	611 (25%)	2398	(24–27%)
Mediums				
Rank	Items	*N* ^*a*^ (%)	Denominator	95%CI
1	Television	1876 (78%)	2391	(77–80%)
2	In-person one-on-one health talk	1849 (77%)	2395	(75–79%)
3	Health information website	1799 (75%)	2398	(73–77%)
	Online consultation with a health professional	1797 (75%)	2395	(73–77%)
5	Radio	1604 (67%)	2388	(65–69%)
6	Recorded online presentation	1420 (59%)	2398	(57–61%)
	Text message	1405 (59%)	2397	(57–61%)
8	Social media	1367 (57%)	2394	(55–59%)
9	In-person face-to-face group presentation	1298 (54%)	2395	(52–56%)
	Booklet	1281 (54%)	2393	(52–56%)
11	Live interactive format online presentation	1264 (53%)	2393	(51–55%)
12	Telephone	1129 (47%)	2397	(45–49%)

^a^Students most preferred (strongly agreed/agreed) cervical cancer education delivery sources, settings and mediums (See supplementary files for further details)

### Preferences for Setting of Cervical Cancer Education

The majority of students (83%) strongly agreed or agreed that hospital was their preferred setting for receiving cervical cancer education. More than 60% of participants strongly disagreed/disagreed that marketplace (63%) and place of worship (75%) were their preferred settings for receiving education (see Table 2). More than four-fifths of the students endorsed at least three items of education delivery settings (see Fig. 1). About 50% of students endorsed a combination of school and home as their preferred setting for education (see Supplementary file).

### Preference for Mediums of Cervical Cancer Education

According to Table 2, more than three-quarters of students frequently endorsed the following mediums for cervical cancer education: television (78%), in-person one-on-one health talk (77%), health information websites (75%) and online consultation with a health professional (75%). Telephone was the least endorsed medium for cervical cancer education. Overall, nearly all students (92%) endorsed at least three items for the delivery medium (see Fig. 1).

## Discussion

This study provides valuable insights for designing cervical cancer education among female high school students in Ghana. Ghanaian adolescents clearly demonstrated preference to receiving cervical cancer education from credible sources via multifaceted and contrasting education delivery mediums suggesting their flexibility to accept a variety of strategies. Most students preferred information from health experts and institutions using either a more individualised approach or anonymised and generic approaches. This study is the first study to explore and report on this range health education of preferences.

### Healthcare Professionals and Organisations were Students Preferred Source of Education

We found that an overwhelming majority of Ghanaian adolescents endorsed a preference to receive cervical cancer education from experts including healthcare professional and health organisations/departments. American students preferred health experts and cancer survivors for cancer education [19]. These findings suggest that students want credible information sources and highlights the need to invest in involving health providers during cervical cancer education. Over half of the students (56%) endorsed teachers and family members (63%), the influence of health education being provided via these channels is noteworthy, especially in low-resourced settings, situations with limited healthcare professionals. There is need to consider how teachers and family members could be supported to accurately supplement other sources of cervical cancer education. Themes emerging from the free-text comments of the survey indicated that students would prefer cervical cancer information from healthcare professionals, teachers and parents. For example, some students provided the following comments: i) “*The health personnels should organize an educative program about the cervical cancer……*” and ii) “*Parents and teachers must advice as all the time from the getting that disease”*. According to the adolescent health literacy framework, teachers play a critical role in enhancing health literacy among adolescents [5].

### Individualised Healthcare-Related and School Settings were Preferred by Students

There was a majority of students endorsing a preference to receive cervical cancer education from a range of individualised care settings (i.e., hospital, home, pharmacy); more than half endorsing school settings; and a minority of students endorsing public settings (i.e., places of worship, marketplaces). In keeping with the large proportion endorsing preferences for receiving education from healthcare professionals, nearly all students wanted to receive education in a hospital setting. While these individualised care settings i) are appropriate, provide some privacy, are very supportive, and comfortable environments to discuss a range of health issues, providing education in healthcare settings is resource-intensive and restricted to either one or small groups of individuals accessing care. School-based health education interventions that are complemented by home-based education may provide a way of meeting most students health education setting preferences in a less resource intensive manner.

### A combination of Home and School-Based Intervention could be Considered for Cervical Cancer Education

The collective finding that a significant proportion of students preferred teachers and family members as their sources of education, home and school as their preferred settings, for education suggest that these options could be considered for low-cost public health campaigns for students. A further analysis found that nearly half of the students (48%) endorsed a preference for a combination of school and home. The free-text comments from the survey supported a school-based education. For example, some students stated that *“I would like some health personnels to come to our school to enlighten us about these infections”*. In line with a recent study, schools were preferred settings for cancer education among American adolescents [19]. In Sub-Saharan Africa, teachers and older siblings were preferred sources of SRH education among adolescents [22]. Supported by previous studies, multicomponent school-based programmes involving home/family where school-based education is reinforced by parents can improve adolescent health knowledge and behaviours including sexual health and preventing smoking [16]. Such programmes, in addition to mass media health documentaries may be expanded to include cervical cancer topics in school curriculums and training teacher, and providing key messages for parents during information sessions. These strategies may elicit conversations at home. Future research should explore strategies to enhance school and home-based cervical cancer education.

### Multiple and Multifaceted Cervical Cancer Education Mediums were Preferred

Students expressed preferences for a wide range of health education mediums and flexibility to accept a variety of these strategies. The 12 assessed cervical cancer education delivery mediums (face-face group presentation, one-on-one health talk, booklet, live interactive online presentation, recorded online presentation, social media, health information website, online consultation with a health professional, telephone, text message, radio, television) were endorsed by between 47 – 78% of students, and nearly all students (92%) endorsed at least three cervical cancer education delivery mediums. The three most preferred mediums, endorsed by at least three-quarters of students, included personalized and individualised (in person or online health talk/consultation), and anonymised and generic (broadcast and digital mass media) mediums. Comments from the students’ survey suggested the need to provide cervical cancer education through one-on-one education, online and social media platforms. For example, the following comments were provided by students: i) *“I am very grateful and happy to be given this opportunity to answer questions like this and i suggest next time it will be a physical or oral interaction with us and also educate us more on health issues”* ii) *“I want to have more information through phone or online chat” and iii) We should continue providing information about the vaccination either on social media, facebook not false information but rather true statement so that those who do not know anything about it can at least express their views or thoughts”.* Personalised one-on-one education has some similarities with students’ accustomed classroom learning, whilst broadcast and credible digital media (i.e., television, radio and internet) has the furthest reach and easily accessible in Ghana; and may potentially explain these flexible and non-mutually exclusive preferences for such different mediums. Future studies could explore whether student’s preferences for mediums of health education cluster together.

### Feasibility of Supporting Student’s Preference for In-Person Cervical Cancer Education

Although in-person sessions could be perceived as resource intensive and non-sustainable, in-person education has several advantages in the Ghanaian setting. These include benefitting people in rural settings who have community health nurses and midwives providing home-based health education [23]. In Ghana, there is a ban on smart devices and phone usage among high school students by the Education Service because of the many distractions of using these devices [24]. Additionally, of the more than half of Ghanaian adolescents who used smart devices (i.e., smartphones and tablets), only 30% of them reported daily use of the internet on their mobile phones [25] and this could perhaps be due to limited internet access in schools [25] or the poor internet connectivity and high internet cost. Therefore, it is important to leverage the existing school-based nursing programme and train school nurses to provide low-cost in-person education to increase accessibility of cervical cancer education. Further, low-cost mass media education could be promoted.

### Feasibility of Supporting Student’s Preference for Anonymised Mass Media Cervical Cancer Education

A significant proportion of students endorsed preferences for broadcast media and credible digital media (i.e., health information website). In agreement with Abraham et al. [20], online videos and educational video games were preferred by more than 80% of American adolescents for cancer education. However, students viewed online information as a potential source of misinformation [19]. In the adolescent health literacy model, Manganello [5] explains that media is part of culture and society, and a pervasive force in adolescent lives, which assists them to access health information. Health websites (including Ghana Health Service) and existing radio and television health programmes and documentaries could be expanded to include microlearning activities about cervical cancer education facilitated by healthcare professionals. During school term, students could be allowed to access online cervical cancer education from these credible websites on school computers. Given the limited internet accessibility, offline versions of online education with prompt updates could improve usability and engagement health education websites.

## Strengths and Limitations

This study provides practical strategies for implementing cervical cancer education among female adolescents using larger sample sizes. However, the findings of this study were restricted to senior high school students aged 16 years and above within government schools in the Ashanti region, reducing the generalizability of the results across other school settings in Ghana. Future research should explore cervical cancer and general health education preferences among students below 16 years and males, and clustering of health education preferences. Additionally, there is the need to examine cervical cancer education preference and associated sociodemographic characteristics.

## Practice Implications

The current study adds to our understanding of how to conduct cancer education to adolescents and young adults and provides valuable insight into the preference of Ghanaian senior high school girls regarding cervical cancer education. It also has broader implications for health and social systems. This information provides practical guidance to health educators about cervical cancer by considering multicomponent approach to promoting cervical cancer messages in Ghana. While doctors and nurses were mostly preferred for education, the shortage of doctors, particularly in regional and rural areas in Ghana [26] warrants the need to focus on equipping nurses. The preference for low-cost interactive online and mass media (i.e., television, health websites and radio) may also lead to increased demand and/or expectations for health education on these platforms. The Ministry of Health could move to capitalise on the expanding nursing workforce in Ghana to provide clear and consistent education about cervical cancer to students through a variety of low-cost interactive online and mass media education strategies. These platforms should create accurate content that increase engagement and usability including high audio and video quality, introducing fascinating facts, visual cues and short informative content. Existing mass media health education programmes could also be leveraged and expanded to include cervical cancer. Additionally, in conjunction with the Ministry of Education, existing school-based nurses could be trained and equipped with materials to provide cervical cancer education to students. While we prepare to train school nurses, it may be also be valuable to extend training to parents and teachers through the Parent and Teacher forums in schools to inform family members about cervical cancer.

## Conclusions

Students preferred a range of multimodal and multifaceted cervical cancer education strategies. These preferences spanned the spectrum from resource-intensive individualised approaches to low-cost anonymous, generic approaches from credible sources and institutions, and suggest a range of education approaches to engage students should be considered. To improve cervical cancer education in Ghana, other low-income settings and resource-constrained situations, relatively low-cost options including broadcast and digital mass media (i.e., television, radio, website) which featured credible healthcare professionals and organisations providing key messages could be considered. Future research should investigate the engagement and cost-effectiveness of E-health strategies for cervical cancer prevention among Ghanaian adolescents including mobile application, interactive online videos and educational games.

### Supplementary Information

Below is the link to the electronic supplementary material.

Supplementary file1 (DOCX 20 KB)

## Data Availability

The datasets used and/or analysed during the current study are available from the corresponding author upon reasonable request.
