# Supplementary material for: Preference For Cervical Cancer Education: A Multisite Cross-Sectional Survey of Female Senior High School Students in Ghana
Source: J Cancer Educ. 2023 Jun 16;38(5):1710–8. doi: 10.1007/s13187-023-02325-5 (PMC10509060; doi:10.1007/s13187-023-02325-5)
Supplement: Supplementary file 1 — Supplementary file1 (DOCX 20 KB) [file 13187_2023_2325_MOESM1_ESM.docx]

**Preference for cervical cancer education: a multisite cross-sectional survey of female senior high school students in Ghana.**

Table S1: Detailed findings of students’ education preference and pattern of missing data

| ***Sources*** | | | | | | |
| --- | --- | --- | --- | --- | --- | --- |
| *Items* | **Strongly disagree** | **Disagree** | **Agree** | **Strongly agree** | **Total** | **Missing data** |
| Doctor | 108 (5%) | 206 (9%) | 1064 (44%) | 1016 (42%) | 2394 | 6 (0.25%) |
| Nurse | 169 (7%) | 310 (13%) | 1362 (60%) | 550 (23%) | 2391 | 9 (0.38% |
| Credible health organization | 147 (6%) | 385 (16%) | 1022 (43%) | 845 (35%) | 2399 | 1 (0.04%) |
| Family members | 246 (10%) | 643 (27%) | 1086 (45%) | 420 (17%) | 2395 | 5 (0.21%) |
| Pharmacist | 197 (8%) | 705 (30%) | 1095(46%) | 388(16%) | 2385 | 15 (0.63%) |
| Teachers | 344(14%) | 720(30%) | 1001 (42%) | 329 (14%) | 2394 | 6 (0.25%) |
| Religious leaders | 401 (17%) | 1025 (43%) | 683(29%) | 277(12%) | 2386 | 14 (0.58%) |
| Traditional leaders | 457 (19%) | 1080 (45%) | 630 (26%) | 227 (9%) | 2394 | 6 (0.25%) |
| Friends | 556 (23%) | 1206 (51%) | 495 (21%) | 129 (5%) | 2386 | 14 (0.58%) |
| Classmates | 611 (26%) | 1178 (49%) | 471 (20%) | 128(5%) | 2388 | 12 (0.50%) |
|  |  |  |  |  |  |  |
| ***Settings*** | | | | | | |
| *Items* | **Strongly disagree** | **Disagree** | **Agree** | **Strongly agree** | **Total** | **Missing data** |
| Hospital | 136 (6%) | 280(12%) | 1108 (46%) | 869 (26%) | 2393 | 7 (0.29%) |
| School during class | 447 (19%) | 984(41%) | 750(31%) | 212(95) | 2393 | 7 (0.29%) |
| School after class | 308(13%) | 812(34%) | 959(40%) | 317(13%) | 2396 | 4 (0.17%) |
| Home | 291(12%) | 663 (28%) | 946 (40%) | 494 (21%) | 2394 | 6 (0.25%) |
| Pharmacy | 265 (11%) | 765(32%) | 1013(42%) | 346(11%) | 2389 | 11 (0.46%) |
| Place of worship | 462 (19%) | 1054 (44%) | 666(28%) | 212(8%) | 2394 | 6 (0.25%) |
| Market place | 628 (26%) | 1159 (48%) | 475(20%) | 136(6%) | 2398 | 2 (0.08%) |
|  |  |  |  |  |  |  |
| ***Mediums*** | | | | | | |
| *Items* | **Strongly disagree** | **Disagree** | **Agree** | **Strongly agree** | **Total** | **Missing data** |
| Television | 164 (7%) | 351(15%) | 1226(51%) | 650(27%) | 2391 | 9 (0.38%) |
| In-person one-on-one health talk | 165 (7%) | 381 (16%) | 1213 (51%) | 636 (27%) | 2395 | 5 (0.21%) |
| Health information website | 190 (8%) | 409 (17%) | 1187 (50%) | 612 (25%) | 2398 | 2 (0.08%) |
| Online consultation with a health professional | 168(7%) | 430 (18%) | 1128 (47%) | 669 (30%) | 2395 | 5 (0.21%) |
| Radio | 201 (8%) | 583 (24%) | 1226 (51%) | 378 (16%) | 2388 | 12 (0.50%) |
| Recorded online presentation | 257 (11%) | 721(30%) | 1076 (45%) | 344 (14%) | 2398 | 2 (0.08%) |
| Text message | 270(11%) | 722(30%) | 1069(45%) | 336(14%) | 2397 | 3 (0.13%) |
| Social media | 284 (12%) | 743 (31%) | 1028 (43%) | 339 (14%) | 2394 | 6 (0.25%) |
| In-person face-to-face group presentation | 344 (14%) | 753 (31%) | 958 (40%) | 340 (14%) | 2395 | 5 (0.21%) |
| Booklet | 305(13%) | 807(34%) | 936(39%) | 345 (14%) | 2393 | 7 (0.29%) |
| Live interactive format online presentation | 343 (14%) | 786(33%) | 982(41%) | 282 (18%) | 2393 | 7 (0.29%) |
| Telephone | 355 (15%) | 913 (38%) | 868 (36%) | 261(11%) | 2397 | 3 (0.13%) |

Table S2: Students’ endorsement of a combination of school and other settings

| *Items* | *N ^a^ (%)* | *Denominator* |
| --- | --- | --- |
| School + Home | 1139 (48%) | 2394 |
| School + Home + Hospital | 1016 (43%) | 2388 |
| School + Home + Hospital + Pharmacy | 722 (30%) | 2379 |
| School + Home + Hospital + Pharmacy + Worship | 419 (18%) | 2374 |
